# Supplementary material for: Are carbon emissions trading and green financial instruments synergistic? -Comprehensive quantitative research based on content analysis
Source: PLoS One. 2024 Mar 7;19(3):e0298601. doi: 10.1371/journal.pone.0298601 (PMC10919601; doi:10.1371/journal.pone.0298601)
Supplement: S1 Data — (ZIP) [file pone.0298601.s001.zip › Data/地级市Z_F工作报告.zip!/─■╧─/╓╨╬└╩╨/└┤╫╘╣1⁄2╓┌║┼í╛┬φ┐╦╩2╛▌═°í┐_2011.docx]

各位代表：

现在，我代表市人民政府，向大会作政府工作报告，请予审议，并请政协委员和列席人员提出意见。

“十一五”及2010年工作回顾

“十一五”时期是我国发展史上极不平凡的五年。面对国内外环境的复杂变化和重大风险挑战，党中央团结带领全国各族人民，紧紧抓住发展第一要务，贯彻落实党的理论和路线方针政策，实施正确而有力的宏观调控，充分发挥我国社会主义制度的政治优势，充分发挥市场在资源配置中的基础性作用，有效应对国际金融危机巨大冲击，举国战胜了历史罕见的低温雨雪冰冻和汶川特大地震等重大自然灾害，成功举办了北京奥运会和上海世博会，保持了经济平稳较快发展良好态势，社会主义经济建设、政治建设、文化建设、社会建设以及生态文明建设和党的建设取得重大进展，国家面貌发生新的历史性变化，谱写了中国特色社会主义事业新篇章！

“十一五”规划是我市的第一个五年规划。五年来，我们与全国人民同喜同悲同拼搏，坚持科学发展不动摇，坚决执行党中央和国务院、自治区党委和政府以及市委各项决策部署，全市上下凝心聚力、团结奋斗，付出了千辛万苦，战胜了重重困难，全面完成了“十一五”各项目标任务，为“十二五”发展奠定了坚实基础。中卫，正以崭新面貌朝着全面小康社会目标奋进！

“十一五”以来，我们取得了重大成就：

综合经济实力上了一个大台阶。

——经济总量实现翻番。2010年，全市地区生产总值达到162亿元，是2005年的2.3倍，年均增长13.6%。人均地区生产总值达到14170元，是2005年的2.1倍，年均增长12.7%。地方财政收入15.21亿元，是2005年的4.3倍，年均增长33.9%。一般预算收入8.25亿元，是2005年的3倍，年均增长24.5%。全社会固定资产投资173亿元，是2005年的3.1倍，年均增长25.3%，5年累计完成固定资产投资490亿元。

——为今后的跨越发展奠定了坚实基础。构筑起产业转移的重要承接地。建成中卫工业园区、中宁工业区、海原新区产业功能区3个工业区和镇罗金鑫园、常乐陶瓷园、宁夏红科技园等6个特色产业园。建成中卫工业园区照壁山水库、供水厂、污水处理厂、固废填埋场、生化氧化塘、蒸发池等重要工业基础设施。全市工业园区总面积由不到10平方公里增加到300平方公里。招商引资取得重大成果。全市入园企业由12家增加到188家。规划项目工业总产值1000亿元以上，其中产值过20亿元的12家，过50亿元的6家。2011年，预计新增工业产值140亿元，全市工业总产值达到310亿元，是2005年的5.4倍。特色农业形成规模。硒砂瓜、马铃薯、枸杞、设施蔬菜、小茴香种植面积分别达到108万亩、113万亩、34万亩、24.7万亩和12万亩。发展高效节水农业示范区10万亩。建成沙漠生态日光温室1200座。“中国枸杞之乡”、“中国硒砂瓜第一市”叫响全国，“中宁枸杞”、“香山硒砂瓜”荣膺中国驰名商标，农产品市场竞争力明显提升。建立了政府偿贷基金制度。通过财政预算模式改革，形成了可支配财力和干大事能力随着财政收入增加而相应增加的良性机制，使融资有了信用保障、发展有了投资保障、偿贷有了资金保障。

人民生活水平和质量上了一个大台阶。

——城乡居民收入明显提高。2010年，城镇居民人均可支配收入13962元，是2005年的2倍，年均增长14.2%。农村居民人均纯收入4315元，是2005年的1.7倍，年均增长11.2%。农村贫困人口由43.5万人减少到21.4万人。

——城乡人居环境发生了令人瞩目的变化。香山机场通航。中宁至营盘水高速公路通车，新建改建公路2875公里，公路通车里程达到5584公里。太中银铁路元月通车。两座6车道铁路立交桥贯通中卫市区南北，铁路以北得以迅速发展。中卫市区建成区由12平方公里扩展到74平方公里。中宁县城由12平方公里增加到24平方公里。海原新区基本建成，县城面貌和功能明显改善。社区休闲园林面积由150亩增加到1.3万亩，水域面积由800亩增加到2.6万亩，集中绿地面积由200亩增加到2.5万亩。香山湖等黄河湿地资源开发保护项目继获我国人居环境最高奖“中国人居环境范例奖”后，新近又荣膺国际人居环境最高奖“迪拜国际改善居住环境最佳范例奖”，是西北第1个、西部第2个、全国第12个迪拜奖，是全球百佳。实施了中卫创建“中国人居环境奖”城市旧街区硬化绿化亮化净化“四化”工程。75%的农村土危平房改造成了坡屋顶抗震砖瓦房。沙坡头区成为“自治区园林城市”。

社会建设上了一个大台阶。

——社会事业长足发展。基本普及九年义务教育，基本扫除青壮年文盲。沙坡头区、中宁县成为“教育强县（区）”，实现了基本普及高中教育，高中阶段入学率由65.6%提高到89.7%，职业教育比重从16%提高到48%。新建中卫中学、中卫职业技术学校等6所中小学、职业学校，在建中卫六中和八小，中卫职业技术学校成为国家中等职业教育改革发展示范学校。基本建成市体育馆、博物馆、图书馆、文化馆、游泳馆、文体中心。市医院120急救中心建成投入使用，新建中医医院康复楼。在全国率先建立重特大病医疗救助制度和中等职业教育免学杂费、书费、住宿费制度。在全区率先建立农村养老补贴和乡村便民公交体系、煤矿视频监控系统和农村垃圾清扫收集转运处理系统。建成企业职工养老保险、失业保险、医疗保险、工伤保险、生育保险“一站式”服务体系。取缔水上非法摆渡。实行网上“一站式”行政服务。

——社会秩序和谐稳定。加强社会治安综合治理和安全生产工作。在全国率先建立了“三调联动”工作机制，全面推行领导接访制度，妥善化解社会矛盾。始终保持高压态势，依法严厉打击各类刑事犯罪。转变警务值勤模式，实行重点部位24小时不间断巡逻，成功侦破了公安部挂牌督办的中宁李小龙涉黑案等大案要案。先后获“社会安全感测评”全区第一、“全国平安优秀市”等称号。严格落实安全生产责任制，依法封堵了所有违法违规矿井及采矿点。在全区率先建立食品药品随机抽检制度，确保了食品药品安全。建立市长信箱制度，3年多为百姓排忧解难6574件，中卫百姓高度信任市长信箱。

各位代表！

2010年是“十一五”规划的最后一年，也是挑战与机遇较突出的一年。我们有效应对国际金融危机后续影响，克服节能降耗限产压力，冷静研判，科学决策，及时出招，扎实苦干，牢牢把握经济工作主动权，圆满完成了市二届人大三次会议确定的各项目标任务。全市地区生产总值达到162亿元，增长13.5%。地方财政一般预算收入达到8.25亿元，增长24.6%。完成全社会固定资产投资173亿元，增长65.6%。实现社会消费品零售总额32.2亿元，增长18%。城镇居民人均可支配收入、农村居民人均纯收入分别达到13962元和4315元，增长9%和12%。

一、致力于工业强市，经济发展后劲增强

工业发展增速加快。完成工业总产值170亿元，增长19.3%；实现工业增加值45.4亿元，增长15.2%。全市规模以上企业完成工业总产值155亿元，增长22.5%；实现工业增加值41.1亿元，增长16%；实现利润6.1亿元，增长74.2%。新建、续建、技改工业项目72个，完成总投资106亿元，增长307%。

招商引资成果十分显著。全年引进招商项目180个，到位资金138.9亿元，增长263%。工业开工项目55个，其中过10亿元产值项目16个。这些项目的引进，使我市初步形成了新能源、新材料、冶金、煤化工、精细化工、造纸6个产业板块。中电投100万千瓦风力发电，国电阿特斯公司10万千瓦光伏发电，金阳公司25万千瓦光伏发电，银阳公司2000吨单晶硅拉棒、2000吨多晶硅铸锭、2亿片切片、10万千瓦电池组件，万隆公司100万吨稀土彩钢板、30万吨镍铁，锦宁公司70万吨铝镁合金，华夏特钢36万吨铬铁，宁钢集团120万吨特钢，宇光公司300万吨洗煤、240万吨焦镁联产，瑞泰公司6万吨硝基氯苯、5万吨氯化苯、1万吨光气等9个产品项目，华御公司5万吨精制苯、6万吨氯化苯等一大批工业项目落地中卫。特别是煤化工和精细化工的产业链，促成了以商招商的大好局面。

能耗与排放明显下降。面对严峻的减排形势，及时出台《节能降耗预警“五定”方案》等4项制度，对全市27家高耗能企业实行阶段性停产或限产，依法关闭15家污染企业。二氧化硫排放量、化学需氧量分别完成自治区下达削减任务的134%和116%。万元地区生产总值综合能耗下降5.2%。

二、致力于打造黄河金岸，最宜居最宜业最具特色美城市建设扎实推进

黄河金岸建设取得新进展。从沙坡头至中宁151公里黄河南北标准化堤防工程全面竣工，151公里滨河大道全线通车。完成151公里滨河大道两侧各110米宽生态湿地绿化工程，植树100万株，恢复湖泊湿地3500亩，整理土地7860亩，生态环境进一步改善。市“五馆一中心”、中宁体育馆、游泳馆基本建成，中国枸杞博物园、杞乡黄河体育中心即将投入使用。香山湖等黄河湿地资源开发保护项目荣获迪拜奖。

人居环境进一步改善。按照“沿黄城市中最宜居、最宜业、最具特色美”的定位，规划、建设和管理中卫市区。深入推进国家园林城市、卫生城市、环保模范城市、中国旅游目的地城市和全国文明城市“五创”工作。完成城市总体规划修编，提高了城市规划前瞻性，城市规划控制面积达到2260平方公里，中卫工业园区、中卫能源化工基地、腾格里沙漠生态屏障等重要功能区纳入了城市总体规划。启动实施创建“中国人居环境奖”城市旧街区硬化绿化亮化净化“四化”工程，完成12条街道硬化绿化整地工程。全市新建、续建城镇建设项目267个，完成投资40.2亿元。市区建成利民路等城市道路3条2.1公里。建成职教公园。完成滨河路、水城路、五环广场亮化工程和景观水系引水工程，横穿市区6公里的景观水系成为一条四季流动的河。中宁滨河新区基础设施基本建成。海原“一县两城，拉动发展”战略顺利实施，老城区基础设施进一步完善，新区供水厂、污水处理厂等市政工程基本完成。

三、致力于农民增收，“三农”工作成效显著

农业经济稳步发展。粮食播种面积250.3万亩，总产5.9亿公斤。实现农业总产值56.5亿元，增长8.4%。鸡、猪、牛、羊饲养量分别达到887万只、116万头、32万头和197万只。建成区、市、县级农田保护示范区6个，粮食高产示范区56个。推广稻田养蟹1.2万亩。引进推广农业新品种133个，新技术42项。新上、技改、扩建农产品加工项目11个，培育农业产业化龙头企业9家。扶持发展专业合作组织52个，全市285个专业合作组织销售农副产品79.8万吨，销售收入15.2亿元。劳务输出17.2万人，创收9.6亿元。足额兑付各类涉农政策补贴5216万元。

特色农业促进了农民增收。新增枸杞4万亩，累计达到34万亩，实现总收入13亿元，杞农人均收入2300元。百万亩硒砂瓜实现总收入11.6亿元，瓜农人均收入2930元。新增设施蔬菜3.9万亩，累计达到24.7万亩，实现产值13.5亿元。发展沙漠农业科技示范大棚及适沙作物1220亩，棚均年收益2万多元。沙漠设施蔬菜2个产品被认证为有机产品。建成永大线旱作节水农业示范区10万亩，栽植枣树等256万株。中宁建成全区最大的供港蔬菜基地。海原建成马铃薯脱毒种薯繁育中心。

农村基础设施建设成效明显。集中实施农村危房改造、农村基础设施建设和城乡环境综合整治“三大工程”，完成总投资13亿元，改造农房10550户，建成“塞上农民新居”示范点15个1936户，综合整治旧村庄57个，配套建设了道路、供排水等基础设施。沙坡头区建立了农村垃圾清扫收集转运处理系统，配置垃圾清运车109辆、垃圾箱11058个，农村告别了千百年垃圾乱弃现象。完成乐台、九塘、迎水桥等农村饮水安全工程，11.8万人的饮水安全得到保障。完成兴仁及永大线节水农业示范项目供水工程，解决了49.5万亩农田用水问题。实施中央财政小农水重点县配套节水、农业综合开发中低产田改造项目，改善9.3万亩农田灌排条件。实施腾格里沙漠整地、永大线高效节水农业用地整理、滨河湿地水源土地整理等项目，整理土地3.9万亩。实施兴仁万人生态移民工程，建设米粮川、高庄等移民安置点5个，226户移民迁入新居，1813户移民2011年6月搬迁。在全市63个贫困村投放互助项目资金1660万元，受益4.8万人。

四、致力于调整结构，第三产业日益繁荣

商贸物流加快发展。完成中卫迎水桥物流园区总体规划。中宁物流园区汽车城投入使用，包兰铁路战略装车点信息平台开工建设。海原新区物流园区基本建成。落实“万村千乡市场工程”、“双百市场工程”等农村市场改造升级项目资金1240万元。建成高崖瓜果蔬菜批发市场。发放中国扶贫基金会惠农扶贫工程项目家电代金券500万元，受益贫困农户1.45万户。扎实推进家电、汽车、摩托车下乡，促进城乡消费。全市社会消费品零售总额达到32.2亿元，增长18%。外贸进出口总额2800万美元。

旅游特色愈加明显。建设旅游项目30个，完成投资2.7亿元。建成沙坡头旅游区黄河文化博物馆、沙漠观光扶梯、大漠露天吧等项目。完成腾格里沙漠湿地旅游项目二期工程，开发金沙岛旅游休闲度假区，建成生态广场、游客综合服务中心和40幢木屋别墅。成功举办首届“沙坡头杯”宁夏国际汽车拉力赛、中国?宁夏（中卫）2010年硒砂瓜暨大漠文化旅游节、黄河金岸国际马拉松赛中卫分赛区健康跑等活动。全年接待游客157万人次，增长9%；实现旅游总收入8.39亿元，增长16%。

房地产业平稳发展。实施房地产开发项目61个，开工建设美利嘉天下、迎宾壹号等商住小区18个，续建正丰香格里、众一山水城等项目43个，施工建筑面积242万平方米，其中沙坡头区146万平方米，增长31%，中宁县96万平方米。提高住房贷款额度，发放住房公积金贷款1.3亿元。商品房销售面积51.9万平方米，其中沙坡头区41.7万平方米，增长69%。全市房地产业实现税收、基金收入8.5亿元，占地方财政收入的56.1%。以房地产业为主的第三产业已成为支柱产业，第三产业占地区生产总值比重为41.2%。

五、致力于改善民生，社会事业全面进步

教育事业进步显著。沙坡头区、中宁县基本普及高中阶段教育，高中阶段入学率由65.6%提高到89.7%。中卫职业技术学校在全区率先实现中等职业教育“三免费”，毕业生当年就业率达96%。六中、八小主体工程完工，为完全解决大班额问题奠定了基础。完成中卫五中、中宁二中、海原红羊中学等194所学校校舍安全改造工程。中宁职教中心、海原职教中心建成投入使用。

科技工作取得进展。西北干旱地区压砂地持续利用关键技术研究示范6个课题通过科技部验收。沙漠有机果蔬集成技术研究示范项目开展10多项设施农业提质增效新技术试验。引黄灌区信息化技术研发集成项目获国家科技支撑计划支持。培育科技型中小企业14家，专利获授权19件。科技特派员引进种养新品种80个，推广新技术19项。争取国家和自治区科技项目27个。创建科普示范县1个、示范乡镇6个、示范村（社区）66个。

文体卫生有改革有进步。完成医药卫生体制改革5项重点工作年度任务。建成市医院120急救中心、中宁县医院住院综合楼、宁安镇卫生院，新建中医医院康复楼、村卫生室27个。在全区市级医院中率先推行电子病历，实现了诊疗信息微机化采集管理。完善食品药品随机抽检制度，推行药品零售企业不良经营行为积分管理制度，监管覆盖率100%。计划生育工作进一步加强，人口出生率、自然增长率分别控制在13.62‰和9.32‰。建成农家书屋146个，举行广场文艺演出100场，在农村放映电影7100场。完成《羊皮筏子非遗文化》、《大麦地岩画》电视纪录片创作摄制。35集电视剧《风雨沙坡头》荣获“影响中国旅游的一部电视剧”金奖。在自治区第十三届运动会和第七届全区少数民族运动会上，我市夺得20金13银10铜的好成绩。

社会保障体系进一步巩固和完善。实施海原县国家级、沙坡头区自治区级和中宁县市级农村养老保险试点，60多万农村居民受益。新型农村合作医疗参合人数87.3万人，参合率95.5%。解决了1.8万名城镇职工及灵活就业人员、5753名关闭破产企业退休人员的养老保险、医疗保险历史遗留问题。城镇居民医保、城镇职工养老、失业、医疗、工伤和生育保险参保人数分别达到8.6万人、7.1万人、3.9万人、7.5万人、4.9万人和4.1万人。新建廉租住房和公共租赁住房5.48万平方米，经济适用住房13.4万平方米。为2247户困难家庭发放廉租住房租赁补贴900万元，廉租住房实物配租487户。拆迁改造、妥善安置城市棚户区727户。

生态环境建设进一步加强。实施腾格里沙漠东南缘防沙治沙造林、生态经济林、大南华水生态林、庄点绿化和农田林网等生态建设工程，植树造林32.1万亩。完成永康、兴仁等5个镇（乡）43个村33.2万亩集体林地确权发证试点工作。沙坡头区迎水桥镇、海原县海城镇被命名为国家级“环境优美乡镇”，迎水桥镇杨渠村被命名为国家级“生态村”。

10件民生实事基本落实。建立农村新型养老保险制度，惠及全市60多万农村居民。扩大特重大病医疗救助范围，为660名城乡特重大病患者发放救助金1214.5万元，为特困患者预借21万元，为4585名城乡低保对象审报住院医疗救助1983.9万元，为9.44万名城乡低保户发放门诊救助和资助参保参合820.3万元。高中阶段入学率达到89.7%，应届初中毕业生升学率达到96.2%。“五馆一中心”基本建成。新建城镇居民安置用房18.88万平方米。新增就业岗位5618个，落实公益性岗位345个，“4050”人员就业159人。兴仁万人生态移民工程建设安置点5个、住房2039套。沙坡头区妇幼卫生“五免一救助”政策惠及群众15628人。沙坡头区农机具免费入户挂牌2200台，免费检验机车940台，免费培训农机驾驶员2200人，免费审验农机驾驶员910人。生态休闲旅游惠民工程惠及市民8.96万人次。开工建设全民创业城、改造东园农贸市场工作未完成。

六、致力于政府效能建设，依法行政和民主法制建设得到加强

政府效能切实提升。出台《中卫市人民政府廉政效能建设九项制度》等7个规范性文件。全面推行项目代建制。精简会议和文件。推行电子政务无纸化办公。严格控制庆典、论坛等活动。严格控制公务接待标准和陪同人员。组织国家工作人员培训18场、考试339场1.5万人次。市政府35个部门（单位）工作人员全部实行手机签到。在全区率先开通了移动远程视频办公系统，实现了利用手机和电脑视频进行远程现场办公，极大地提高了行政效率和质量。规范政务服务中心各项工作，251项行政审批事项和58个收费项目全部进入中心“一站限时”办理，全年共受理41501件，办结率100%。推行市长督办副市长重点工作制度，对“A类重点工作”在市政府常务会议上督办。完善市长信箱制度，启用市长信箱印章，规范受理、办理和答复程序，2010年解决群众合理诉求2869件。百姓的诉求在7天内得到答复，市区每5个家庭中有1个家庭得到过市长信箱帮助，百姓的非涉法诉求98%以上通过市长信箱得到解决。中卫市特重大病医疗救助、中等职业全免费教育、农村环境卫生管理长效机制、国家工作人员法律与专业三年培训规划等15项工作被自治区人民政府确认为2010年度创新性工作。

民主法制不断加强。自觉接受人大法律监督和政协民主监督，办理人大议案、建议48件，政协提案147件，办复率100%。深入贯彻党的民族宗教政策，各族人民和睦相处。加强与工会、共青团、妇联等人民团体和民主党派、工商联的联系。强化行政监察和审计监督，开展了工程建设领域突出问题、机关事业单位“小金库”专项治理，完成部门（单位）财政预（决）算审计10项、领导干部经济责任审计15项、民生资金审计12项、政府建设项目决算审计16项，审减工程造价1190万元，审减率8.6%，查处国有企业违规资金1066万元。圆满完成第八届村委会换届选举。“五五”普法通过自治区验收。

社会大局和谐稳定。扎实推进“三项重点工作”，着力化解社会矛盾，在全市40个镇乡设立综治工作中心，健全完善“三调联动”机制，共受理群众来信来访5530件，同比下降28.7%，自治区交办的15件涉法涉诉信访案件全部化解。促进社会管理创新，市区重点单位、重点部位电子监控率达到100%，全市挂牌的32个社会治安重点地区得到有效整治。在全区社会治安综合治理工作评比中获五市第一名，沙坡头区被评为2010年度“平安市（县、区）”。严厉打击刑事犯罪，破获刑事案件1786起，百名民警破获刑事案件数188.3起。治理道路交通黑点21个，确保群众出行安全。加大文化市场监管力度，网吧、歌舞娱乐等文化市场健康有序。加强矿产资源管理，出台《中卫市煤矿整顿关闭安全管理九条措施》，封堵了所有违法违规煤井。“平安中卫”建设扎实推进，人民群众安居乐业。

第六次全国人口普查顺利开展，人口登记工作圆满结束。成功举行地震、消防、交通安全等应急演练。国防教育、双拥共建、民兵预备役、人民防空、金融、税务、统计、物价、市志、档案、外事、侨务、保密、老龄、残疾人、邮政、通信、气象等各项工作取得了新成绩。

各位代表！

回顾五年的历程，我们深切地感受到，做好政府工作，必须深入贯彻落实科学发展观，促进经济社会又好又快发展；必须坚持以人为本，促进民生改善和社会和谐；必须扎实推进新型工业化，增强经济实力和发展后劲；必须扎实推进社会主义新农村建设，破解“三农”难题；必须扎实推进城乡一体化，解决发展不平衡问题；必须大力发展县域经济，加快建设全面小康社会；必须深化改革开放，增强经济发展的生机和活力；必须大力建设法治型、效能型、廉洁型、服务型政府，提高领导科学发展的能力和水平。

五年艰辛创业，成绩来之不易。这是自治区党委、政府和市委正确领导的结果，是市人大、政协大力支持的结果，是社会各界和衷共济的结果。这些成绩，凝聚着全市广大干部群众的辛勤汗水和智慧。在此，我代表市人民政府，向全市人民，向人大代表、政协委员，向各民主党派、工商联、无党派人士、人民团体，向驻卫部队、武警官兵，向所有关心、支持和参与中卫建设与发展的朋友们，表示崇高的敬意和衷心的感谢！

在肯定成绩的同时，我们也清醒地认识到还存在着许多问题和困难。一是我市还处在社会主义初级阶段的较低层次，爬坡追赶任务重难度大。二是面对严峻的节能任务，招商引资和工业发展难度更大任务更艰巨。三是全市还有一半人生活在干旱地区，有21.4万贫困人口，有8.46万人生活在极度干旱缺水不适宜人居地区，生态移民与反贫困任务十分繁重。四是消费市场小，灌区耕地少，山区条件差，城乡居民增收难。五是国家工作人员履职能力和工作作风参差不齐，行政效能建设有待进一步加强。我们将采取有效措施不断解决这些问题。

关于“十二五”规划

各位代表！

“十二五”规划纲要已提请大会，请予审议，并请政协委员和列席人员提出意见。

“十二五”时期，是全面建设小康社会的重要时期，是深化改革开放、加快转变发展方式的攻坚时期，更是我市加快发展、缩小差距、奋力跨越的关键时期。我们必须面向全面建设小康社会目标，切实抓住大有作为的重要战略机遇期和新一轮西部大开发战略机遇，把中卫交通、土地、水利三大优势转化成为发展优势，努力把中卫建成“第二宁东”、宁夏第二个“最宜居最宜业最具特色美”城市、“疆煤进宁”和“蒙煤进宁”基地、产业转移的重要承接地，圆满完成“十二五”规划各项目标任务。

指导思想是：高举中国特色社会主义伟大旗帜，以邓小平理论、“三个代表”重要思想为指导，深入落实科学发展观，认真贯彻党中央、国务院各项决策，坚决执行自治区党委、政府和市委工作部署，深入推进西部大开发，科学实施“十二五”规划，致力于加快“引入经济”与“内生经济”双增长，致力于加快经济发展方式转变和经济结构调整，致力于加快生态文明建设和城乡人居环境改善，致力于推进改革开放和服务型政府建设，致力于改善民生、促进社会和谐稳定，着力提升传统产业，大力发展优势特色产业，重点培育战略性新兴产业和现代服务业，促进经济社会更好更快发展，努力把中卫建成一个经济持续繁荣、生活舒适便捷、景观优美宜人、社会和谐稳定、文化丰富深厚、具有公共安全的现代城市。

发展目标是：全市地区生产总值达到350亿元，年均增长14.6%。人均地区生产总值达到29000元，年均增长14.1%。地方财政一般预算收入达到16.8亿元，年均增长18.8%。城镇居民人均可支配收入达到24600元，年均增长12%以上。农村居民人均纯收入达到7600元，年均增长12%以上。全社会固定资产投资5年累计达到1774亿元，年均增长25%。社会消费品零售总额达到68亿元，年均增长16%。人口自然增长率控制在10.8‰以内。城镇化率提高到50%。森林覆盖率提高到17.5%。全面完成自治区下达的节能减排任务。

主要任务和重点项目是：一是着力发展工业。重点建设3个千亿元产值工业区。启动中卫能源化工基地千亿元产值工业区建设，在甘塘地区450平方公里范围内规划建设生态能源化工工业城，把甘塘地区建成国家重要的生态屏障、重要的能源化工基地和著名的沙漠旅游景区。重点发展电力能源、煤化工产业，规划总投资2600亿元，总产值1700亿元。主要是年产煤制油1000万吨、火力发电2000万千瓦。扎实推进中卫工业园区、中宁工业区建设千亿元产值工业区各项工作。建成高新技术产业园和多金工业园。着力打造6个工业基地。改造提升传统优势产业。加快电力设施建设。全面提高信息化水平。加大招商引资力度。大力发展民营经济。二是加快发展现代农业。建设50万亩枣瓜间作高效节水农业。着力提升优势特色产业。加快发展农副产品精深加工。加快推进农业产业化。夯实水利基础设施。扎实推进新农村建设。实施新型生态移民工程，对极度干旱不适宜人居地区8.46万人实行教育培训移民、城镇就业移民、发展型生态（农业、林业）移民和养老保障移民。三是提高城市建设与管理水平。加快建设沿黄城市带。着力打造宁夏第二个“最宜居最宜业最具特色美”城市，创建国家园林城市、卫生城市、全国文明城市、“中国人居环境奖”城市。大力发展房地产业。加快中宁县城和海原新区建设步伐。加快小城镇建设。四是加快发展现代服务业。全面打造旅游品牌，建设宁夏国际沙漠博览园，开发沙坡头南岸半岛景区景点。创建中国旅游目的地城市。加快发展商贸流通，建设沙坡头区大型农产品综合贸易市场、海原新区国家供销中心农副产品批发市场。大力发展新型服务业，发展村镇银行、土地银行、民营金融服务机构、信用担保中心。加快发展现代物流业，建成迎水桥物流园区、中宁物流园区和海原新区物流中心。五是加强生态建设和环境保护。加快推进生态建设，实施重点生态工程建设和重点生态区综合治理项目。加大环境治理和保护力度。切实加强节能降耗。创建国家环保模范城市。六是加强综合运输体系建设。加快建设铁路交通基础设施，建成中卫至阿拉善盟苏宏图至乌力吉口岸铁路，实施包兰复线、宝中复线、甘武复线中卫段扩能改造，改扩建中卫火车站和中宁火车站，新建海原火车站。进一步加强公路交通基础设施建设，建成海城至海原新区高速公路，新建中卫下河沿黄河公路大桥，拓宽改造中卫黄河公路大桥和中宁黄河公路大桥，新建改建各类等级公路4161公里。扩建香山机场。七是全面发展社会事业。优先发展教育事业，建设中卫第二职业技术学校。实施人才强市战略，增强科技创新能力。繁荣文化体育事业。提高医疗卫生服务水平，建设救护康复体检中心。切实稳定低生育水平。努力扩大就业。健全社会保障体系。加强安全生产和防灾减灾。八是积极推进精神文明建设和民主法制建设。推进精神文明建设。大力发展社会主义民主。加强社会管理。九是积极推进体制改革。推进行政管理体制改革。深化民生保障体制改革。协调推进城乡综合改革。稳妥推进社会服务改革。加快医药卫生体制改革。完善投融资体制改革。深化财税金融体制改革。

2011年工作安排

各位代表！

2011年，是“十二五”起步之年，也是实施新一轮西部大开发战略的第一年。迈好第一步，至关重要。2011年的主要预期目标是：完成地区生产总值185亿元，增长13.2%。人均地区生产总值达到16000元，增长14%。完成工业增加值55亿元，增长20%。地方财政一般预算收入10.1亿元，按可比口径增长18.9%。全社会固定资产投资218亿元，增长26%。社会消费品零售总额达到37亿元，增长14.9%。城镇居民人均可支配收入15358元，增长10%。农村居民人均纯收入4833元，增长12%。城镇新增就业7500人，城镇登记失业率控制在4.3%以内。人口自然增长率控制在10.9‰以内。全面完成自治区下达的节能减排任务。

一、突出发展工业，加快推进新型工业化

着力把中卫建成产业转移的重要承接地。启动中卫能源化工基地千亿元产值工业区建设。完成中卫能源化工基地总体规划评审，开工建设神华宁煤2×100万千瓦火电项目，力争开工建设国电4×100万千瓦火电项目，建设道路、水库等基础设施。加快中卫工业园区、中宁工业区发展。扎实推进两个工业区项目引进、建成投产、达产达效等建设千亿元产值工业区各项工作，完善基础设施，提升承载功能。着力打造6个工业基地。利用中卫交通枢纽优势、地处“西煤东运”中间地带的区位优势、水利和土地资源优势，集约发展煤化工、煤制油、煤变电、天然气制甲醇、合成氨等产业，打造能源化工基地。加快发展铝镁合金、稀土彩钢板等合金材料、光伏发电材料、新型建筑装饰材料、石膏制品和其他新材料，打造新材料基地。积极承接东部地区陶瓷产业转移，扩大建筑陶瓷生产规模，加快发展精细陶瓷，打造陶瓷工业基地。加快发展特种钢、金属锰等冶金产业，打造黑色和有色金属深加工基地。加快建设香山、甘塘地区风力发电和腾格里沙漠南缘、南山台、香山罗泉地区光伏发电项目，打造新能源与战略性新兴产业基地。加快建设中冶美利纸业林纸一体化二期工程，全面提升造纸行业生产规模和技术装备水平，打造造纸产业基地。

抓紧抓实抓好招商引资。下大力气、卓有成效地开展招商引资工作。紧紧盯住耗能低、排放少、产值高、技术先进的项目，围绕投资意向、合同签约、落地开工、达产达效4个招商环节，落实责任单位、责任人、责任事项、责任时限、责任处罚“五定”工作制度，达成意向一批、签约合同一批、落地开工一批、达产达效一批，让大企业好企业源源不断地落地中卫。在建55个项目上半年建成达产。即确保宇光公司240万吨焦镁联产、300万吨洗煤，银阳公司2000吨单晶硅拉棒、2000吨多晶硅铸锭、2亿片切片、10万千瓦电池组件，瑞泰公司6万吨硝基氯苯、5万吨氯化苯、1万吨光气，万隆公司100万吨稀土彩钢板等项目达产达效。新增工业产值140亿元，使全市工业总产值达到310亿元。招商引资到位资金100亿元。即开工项目29个，协议投资300亿元，计划产值280亿元。确保神华宁煤2×100万千瓦火电，中电投2×35万千瓦热电联产，天津亚东萘系列产品，重庆万利来氨醇及甲醇蛋白，锦宁公司30万吨铝原板、70万吨铝镁合金，海原热电联产镁合金工业园、30万千瓦风电等项目年内开工建设。签订投资意向项目25个。协议资金300亿元以上，计划产值超过250亿元。主要是淮化集团30万吨强硝酸、30万吨合成氨，多维药业4万吨复合维生素，名泰公司30万吨变性淀粉，利安隆公司8000吨紫外线吸收剂、7000吨抗氧剂等项目。

二、突出打造黄河金岸，加快推进城乡一体化

加快黄河金岸建设。围绕建设宁夏第二个“最宜居最宜业最具特色美”城市目标，开工建设宁夏国际沙漠博览园、金沙岛休闲度假区二期工程等一批黄河金岸标志性建筑。建设滨河大道南北两侧河道整治及护堤工程共13处。建设滨河路、香山公园、应理湖等地段路灯景观照明，向东延伸景观水系6公里，形成新的城市景观。开工建设中卫火车站改扩建项目和中卫工业园区铁路专用线。建成中国枸杞博物园、杞乡黄河体育中心。完成中宁南河子公园扩建工程，开工建设中宁五星级酒店和石空滨河公园，努力把中宁县城打造成沿黄城市中的“金岸明珠”。充分发挥海原新区经济增长极的作用，全面完成新区二期道路、供排水、学校、医院和38家单位办公楼建设，加快新区景观水道、污水处理厂、图书馆、体育馆、花儿艺术馆等工程建设，把海原新区打造成西北最漂亮的山城。

建立新市民（农民工市民化）保障制度。梯度放开农民工户籍。辖区内有合法固定住所，有固定经营场所从事二、三产业或在本辖区企事业单位签有人力资源和社会保障部门认定的劳动合同者，以及在我市职业学校就读的农村学生均可申报登记为城镇常住户口。凡取得中卫城镇户籍，且在中卫工作或从业三年以上者，可申请政府公租房或廉租房，同时按相关政策可享受就业、就学、养老、医保、低保等待遇。建设新市民保障住房。2011年沙坡头区开工建设公租房和廉租房40万平方米，竣工20万平方米。中宁县建成公租房和廉租房1.7万平方米。海原县建成公租房和廉租房0.3万平方米。主要用于安置新市民。

加快中卫市区改造与建设。完成中卫市区城市总体规划修编。科学划定城市功能区，拓展城市规划面积，将450平方公里的中卫能源化工基地、7平方公里的高新技术产业园和中卫工业园区及其东扩区70平方公里等区域纳入城市总体规划，城市总体规划控制面积达到2260平方公里。一次规划、分期改造“城中村”。用2年时间，通过农房改造、商业开发等方式，对老城区所有“城中村”全部进行拆迁改造，妥善安置拆迁户，彻底改善老城区面貌。2011年完成2个点，拆迁改造2000户。实施创建“中国人居环境奖”城市旧街区硬化绿化亮化净化“四化”工程。用2年时间完成老城区所有道路，即58条街道“四化”改造，为创建“中国人居环境奖”城市创造条件。2011年完成中山路、鼓楼东街、应理南北街、怀远南北街、文昌南北街、长城街、蔡桥路、文萃南北路等12条街道“四化”工程，并完成剩余46条街道“四化”整地工程。对市区中心配电室及沙坡头大道等10条主要交通干道实施路灯节能改造，改造红太阳广场景观照明，新增三合路等地段路灯照明。扎实推进“五创”工作。继续抓好创建国家园林城市、卫生城市、环保模范城市、中国旅游目的地城市和全国文明城市工作。

三、突出发展现代农业，推进广大农村向全面小康迈进

大力发展高效特色农业。粮食播种面积稳定在196万亩，总产量5.3亿公斤以上。全面推行设施农业提质增效12项措施，促进高效设施农业模式化种植。硒砂瓜、马铃薯、设施蔬菜、枸杞和小茴香稳定在100万亩、110万亩、24.7万亩、34万亩和12万亩。实施兴仁综合供水工程续建项目、永大线高效节水农业二期供水等水利建设工程。完成永大线20万亩枣瓜间作节水农业示范项目建设。建成中宁红梧山万亩现代有机枸杞节水示范基地和海原高崖乡自治区级旱作高效节水农业示范区。充分利用现有的50万亩生态林业资源，探索发展以养鸡为主的林下养殖业，全市鸡饲养量达到750万只。

实施新型生态移民工程。贫困人口小康才能全面小康。坚持市级负责、县区联动，整体搬迁、分类安置，统一规划、一以贯之原则，用5年时间对极度干旱不适宜人居地区的8.46万人口全部移民。其中：对1.4万适学人口实行教育培训移民，对3.78万适业人口实行城镇就业移民，对2万适农人口、0.36万适林人口实行发展型生态移民，对0.92万老弱病残人口实行养老保障移民。通过提高人均耕地、增加城镇就业、建设公共设施、加强技能培训、提供社会保障、梯度放开户籍，让贫困农民告别“贫困的恶性循环”。2011年完成2500套移民住房建设，移民3万人。其中：教育培训移民5000人、城镇就业移民1.5万人、生态农业移民5000人、养老保障移民5000人。移民迁出区全部退耕。

加快新农村建设。继续实施农村危房改造、农村基础设施建设和城乡环境综合整治“三大工程”，改造农村危房3000户，新建“塞上农民新居”示范点13个1360户。全面启用农村垃圾清扫收集转运处理系统，让农村遍地的垃圾从此消迹，再现“塞上江南”农村美好的自然风光。继续对集中连片50户以上的村庄进行基础设施配套建设，硬化巷道、铺设供排水主管道。加快建设大庄点、大小区，让农民住进小区。实施沙坡头区宣和、永康等5个集镇和中宁石空镇基础设施改造建设工程。新建改造农村公路178公里。

四、突出培育第三产业，不断优化产业结构

重点发展旅游产业。建成沙坡头旅游区观光扶梯、南区度假村等项目。深度开发腾格里湿地公园，完善游客综合服务中心等配套设施。围绕“黄河古城、浪漫沙都、花儿杞乡”旅游品牌，打造“一区”、“两寺”、“三线”、“四湖”旅游精品线路。加强宣传促销，拓展客源市场，全市旅游人数达到165万人次，旅游总收入突破9亿元。

重点发展房地产业。全面落实促进房地产业健康发展的各项政策措施，优化住房供应结构，鼓励住房供应，政府确保提供20万平方米保障住房。鼓励房地产企业投资开发，促进有效需求，进一步稳定房价。全市施工建筑面积200万平方米以上。全年发放住房公积金贷款1.4亿元。进一步规范国有土地供应，防止违法违规用地、拖欠土地出让金、土地闲置等问题发生，确保国有土地效益最大化。

重点发展商贸物流业。开工建设重点物流园区。启动建设迎水桥物流园区，建成中宁战略装车点、海原物流园区。支持企业实行连锁经营、物流配送、电子商务等，促进流通现代化。加快市场建设。继续实施“万村千乡市场工程”和“双百市场工程”，开工建设中卫市大型农副产品批发市场，建成海原县国家供销中心农副产品批发市场及配送中心等项目。积极争取中小企业国际市场开拓项目和西部外贸区域协调发展项目等资金，扶持重点外贸企业发展。实现外贸进出口总额3000万美元。

五、突出保障改善民生，全面发展社会事业

办好6件民生实事。建立新市民（农民工市民化）保障体系。实施新型生态移民工程。实施创建“中国人居环境奖”城市旧街区“四化”工程，综合整治农村环境。扩大职业教育覆盖面。新建市救护康复体检中心。建设全民创业孵化基地。

进一步办好人民满意的教育。继续把教育摆在优先发展的战略位置，加快发展学前教育，着力推进义务教育均衡发展，进一步提升普通高中教学质量，大力发展职业教育。深入推进素质教育，全面提高教育教学质量。继续实施中小学校舍安全工程，改扩建中卫三中、中卫三小、兴仁小学，中宁三中、中宁二小，海原树台中学、关庄中学等学校。加大第二轮学校布局调整力度，撤并沙坡头区川区教学点。实施中卫职业技术学校二期工程，争取升格为职业技术学院，中职毕业生当年就业率达到96%以上。建设中卫第二职业技术学校。海原县争取创建“教育强县”。

进一步发展科技事业。抓好硒砂瓜、沙漠日光温室的提质增效工作，年内引进新品种20个，示范推广新技术5项。在新能源、新材料、先进装备制造、生物制药等领域积极开展科技研究，争取有3个以上工业项目列入国家级科技创新项目计划。抓好农村信息化网络建设，重点培育8个信息服务示范点。完善科技特派员管理机制，全市科技特派员总数突破400人，新培育科技特派员创业企业2家以上。

进一步健全社会保障体系。全面推进社会保险基金征管体制改革，建立健全城乡统筹养老保险体系。完善新型农村社会养老保险制度，参保率达到70%以上。新型农村合作医疗参合率达到90%以上。城镇居民基本医疗保险参保人数达到8万人。城镇职工养老、失业、医疗、工伤和生育保险参保人数分别达到7.1万人、3.5万人、7.2万人、4万人和4万人。开展农民工职业技能培训1万人、城镇各类人员职业技能培训2500人，职业技能鉴定6000人。确保农村劳动力转移就业16.5万人，实现劳务收入8.8亿元。实施“中小企业成长工程”，建立创业基金。建设全民创业孵化基地，培育小企业300个、小老板300个，创造新岗位3000个，创新开展“1+X”创业能力培训900人。帮助400名城镇下岗失业人员实现再就业，帮助400名高校毕业生就业。进一步完善特重大病医疗救助制度和城乡居民最低生活保障制度，加强救灾救济工作。开工建设经济适用住房12.7万平方米，廉租房和公租房40万平方米，竣工20万平方米。

进一步提高公共卫生服务保障能力。全面落实中卫市医药卫生体制年度改革任务，加强医护人员管理与业务培训，提高医疗卫生水平。改革医护人员工资待遇，不断提高医疗护理水平。实施市人民医院精神病科楼和兴仁中心卫生院改扩建等项目，开工建设市救护康复体检中心。加强预防接种、传染病网络直报等疾病预防基础工作，将重大传染病疫情隐患消灭在萌芽状态。加大食品餐饮企业、加工摊点卫生监管力度，实现卫生监督全覆盖，确保群众饮食卫生和安全。深入推进计生民生、优生促进、人口信息化三项工程，切实稳定低生育水平。

大力发展文体等社会事业。“五馆一中心”投入使用。新建村级农民体育健身工程15个、农家书屋300个。争取非物质文化遗产《羊皮筏子制作技艺》进入国家级保护名录。完成第三次全国文物普查工作。实施文化信息资源共享、文化下乡、文化精品三项工程，完成山区1800户常住农户广播电视户户通工程。沙坡头区村、社区每月放映1场电影。组织文艺团体下乡10次以上。开展大型广场文化演出30场次以上。

六、突出改革创新，不断理顺发展体制机制

深化行政管理体制改革。进一步转变政府职能，做到情况不明不决策，只有一个方案不决策，未经可行性论证不决策，情绪不稳不决策，重大政策未经试点不决策。全面推行项目代建制、电子政务无纸化办公、国家工作人员培训和考试、市政府各部门（单位）工作人员手机签到、市长督办A类重点工作、行政审批事项和收费项目政务服务中心“一站限时”办理等廉政效能建设九项制度，切实提高行政效能。减掉8%行政审批项目，逐步实现“法无禁止即许可”。

深化农村综合改革。稳步推进农村土地经营权流转和适度规模经营，大力发展农业专业合作组织。创新农业支持保护制度和现代农村金融制度，积极争取村镇银行、贷款公司试点，筹建农村信贷担保公司，加大信贷支农力度。改革集体林权制度。推进户籍制度改革。全面推行“村财民理乡监管”，完善村民民主管理。

加强财税管理。继续推进政府财政向发展财政、民生财政、公共财政转变。加大对成长型、税源型企业扶持力度，培育壮大支柱财源，增加可支配财力。加强财政监督检查和审计监督，完善财政资金审批制度，加强政府采购、国库集中支付管理，提高资金使用效益。

七、突出社会和谐稳定，加强民主法制建设

深化平安创建活动。抓住人民群众最关心的公共安全、权益保障、社会公平正义问题，深化社会矛盾化解、社会管理创新、公正廉洁执法“三项重点工作”，建立健全科学的预防化解矛盾机制，构建立体化社会治安防控体系。着力突破人口服务管理中的难点问题，加强对新经济组织、新社会组织和新兴媒体的服务管理。加强政法队伍和政法机关基层基础建设。推进司法改革，促进司法公开。依法打击犯罪，筑牢维护社会和谐稳定的第一道防线。深入开展煤矿和非煤矿山、道路交通、建筑施工、危险化学品、人员密集场所等重点行业和重点领域安全隐患排查治理，预防较大事故、重大事故、特大事故发生。

加强民主法制建设。自觉接受人大及其常委会的法律和工作监督，主动接受政协的民主监督，办好人大代表议案、建议和政协委员提案。深入贯彻党的统一战线和民族区域自治政策。依法管理宗教事务，建立宗教场所文化图书室5个。广泛开展双拥共建、精神文明建设、思想道德建设、民族团结进步创建活动。扎实推进“六五”普法。

办快办好群众来信来访。更好地发挥市长信箱了解民情、为民解难的高效渠道作用，让群众合理诉求一件不搁地得以解决。严格落实各级领导干部信访接待日制度，更加注重从源头上预防信访问题，把民事纠纷化解在当地，不断加固社会和谐稳定的基础。

八、突出政府效能建设，提升领导科学发展的能力

建设法治型政府。规范行政执法行为，加强对工程招投标、政府采购、国有资产转让等公共资源管理权力的监督。完善听证、论证和专家咨询制度，重大决策做到问政于民、问计于民、问需于民，提高决策的科学化民主化水平。继续实施《中卫市国家工作人员法律与专业三年培训规划》，建设政治坚定、业务精通、作风优良的国家工作人员队伍。

建设效能型政府。严格执行《中卫市行政审批事项目录和行政审批事项“一站限时”办理规定》、《中卫市行政监察建议办法（试行）》和《中卫市人民政府严格逐级请示报告工作制度》，着力提高领导干部谋划发展、统筹协调、公共服务、社会管理、维护稳定的能力。大力精简会议和文件，严格控制庆典、论坛等活动以及公务接待标准和陪同人员，降低行政成本。严格执行《中卫市人民政府市长督办副市长重点工作督办办法》，在每次市政府常务会议上督办重点工作，力保重点项目落到实处。

建设廉洁型政府。贯彻落实《廉政准则》。严格执行党风廉政建设责任制，加强对政府及其组成部门主要领导干部经济责任、专项资金和重大投资项目审计监督。进一步完善国有资产管理、工程建设和政府采购等重点领域的监管制度，确保行政权力在阳光下运行。坚持艰苦奋斗、厉行节约。加强政风行风建设，坚决依法查处损害群众利益的行为。

建设服务型政府。建成政府政务信息、移动办公、数字城市和公共服务4个平台，完善中卫信息港网站，全面启用市长视频指挥系统、远程移动办公系统、视频会议系统、车辆定位系统、数字城市管理系统，建成财税管理、待遇支付、社会保障等“一卡通”服务平台和农村信息化平台，逐步实现行政管理数字化、公共服务智能化。

各位代表！

中卫是个宜居宜业的好地方，后发优势凸现，前途十分光明。让我们更加紧密地团结在以胡锦涛同志为总书记的党中央周围，在自治区党委、政府和市委的坚强领导下，坚定地走科学发展道路，一心一意谋发展，竭尽全力促跨越，为建成全面小康社会努力奋斗，以优异的成绩迎接中国共产党成立90周年！
